# Bariatric Surgery: Consequences on Functional Capacities in Patients With Obesity

**DOI:** 10.3389/fendo.2021.646283

**Published:** 2021-04-01

**Authors:** Aline Reinmann, Simone Chantal Gafner, Roger Hilfiker, Anne-Violette Bruyneel, Zoltan Pataky, Lara Allet

**Affiliations:** ^1^ Geneva School of Health Sciences, Haute Ecole Spécialisée de Suisse Occidentale (HES-SO) University of Applied Sciences and Arts Western Switzerland, Geneva, Switzerland; ^2^ Valais-Wallis School of Health Sciences, HES-SO, University of Applied Sciences and Arts Western Switzerland, Valais, Switzerland; ^3^ Department of Medicine, University Hospitals of Geneva, University of Geneva, Geneva, Switzerland

**Keywords:** obesity, functional capacities, strength, quality of life, balance

## Abstract

**Introduction:**

Bariatric surgery leads to loss of fat and fat-free mass (FFM). To preserve FFM it is recommended that weight loss interventions are accompanied by physical activity. However, it remains unknown if functional capacities spontaneously improve after a substantial weight loss. Study’s aim was to assess the effect of bariatric surgery on strength, functional capacities and quality of life of participants with a body mass index (BMI) ≥ 35 kg/m^2^.

**Method:**

Anthropometric parameters (weight, BMI, waist circumference), physical functions (quadriceps strength, walking capacity, spatio-temporal gait parameters, dynamic balance, lower limb power) and quality of life were assessed before and at three months after the bariatric surgery in 33 individuals who did not follow any physical activity program.

**Results:**

The anthropometric parameters, quality of life and three functional abilities (walking capacity, normal gait speed and lower limb power) improved significantly three months post-surgery. In contrast, fast gait speed, absolute strength, normalized strength, and dynamic balance did not improve.

**Discussion:**

A massive weight loss should be accompanied by an exercise program aiming to maintain FFM and to enhance muscle strength and balance. Such a program might also enhance functional capacities and help to sustain the weight loss over time.

## Introduction

Obesity is a complex and multifactorial disease known to increase the risk of cardiometabolic complications [e.g. hypertension, heart disease, type 2 diabetes mellitus, dyslipidemia, arthritis, obstructive sleep apneas and cancers (1)].

To counteract the obesity-related complications and to improve the quality of life in people suffering from obesity, a weight loss of 5-10% is often recommended (2, 3). Bariatric surgery is a treatment approach to be considered when standard weight loss therapy by lifestyle modifications have failed (4). The food restriction and the malabsorption of nutrients induced by surgery lead to a more important weight loss than with lifestyle interventions (5). However, a bariatric surgery not only leads to loss of fat mass but also to loss of muscles and bone mass, the so-called fat-free mass (FFM). The average loss of FFM in percentage to the total weight loss following bariatric surgery is estimated to be 31.3 (± 12.2)% (6). This decrease of FFM has health related consequences as it increases patients’ fracture risk, affects muscle strength and individuals’ functional capacities (7). In addition, the loss of FFM negatively impacts the total energy expenditure and thus increase the risk of weight regain (8). In summary, the preservation of FFM is crucial as its loss is related to different health problems and even associated with an increased all-cause mortality (9).

To preserve FFM it is recommended that weight loss interventions are accompanied by physical activity (PA). More specifically, the loss of FFM can be limited if the weight loss, whether as a result of lifestyle interventions (10, 11) or bariatric surgery (7), is accompanied by a supervised resistance and aerobic exercise program (6). In addition, PA helps to sustain the weight loss over time (7, 12). According to Chaput et al. (12), possible mechanisms explaining why PA prevents from regaining weight are its influence on the resting metabolic rate and the daily energy expenditure. Another possible explanation was that PA promotes a better adherence to energy-restricted diets and lifestyle counseling (12).

Despite the benefits of PA, people with obesity are known for not being active enough (8, 13), neither before nor after a bariatric surgery. They often fail to reach the recommended levels of at least 150 minutes of moderate to vigorous PA per week (14). Besides the individual attitudes toward PA (13) and the self-presentational concerns (e.g. concern about appearance), the impaired functional capacities related to obesity such as a slower gait speed, balance deficits, less powerful legs in relation to the body weight and a poorer endurance, may further explain the sedentary behaviors of people with obesity (15) and may influence the quality of life of individuals (16). However, it remains unknown if functional capacities automatically improve after a substantial weight loss or if they rather deteriorate if no specific exercise program aiming to maintain FFM and train functional capacities accompanies the weight loss. We were thus interested to assess if different functional parameters improve due to the weight loss alone or if they deteriorate, due to the induced loss of FFM after bariatric surgery.

The aim of the present study was to assess the effect of a massive weight loss after bariatric surgery on muscle strength, patients’ functional capacities (standing up from a chair, dynamic balance, gait, walking capacity) and quality of life, parameters required to be physically active. We hypothesized that some of these functions (balance, gait and walking capacity) as well as quality of life improve, thanks to the weight loss alone. However, we further hypothesized that other parameters, such as muscle strength and standing up from a chair will not improve due to weight loss alone. A confirmation of our hypothesis would underline the necessity to accompany these patients in their weight loss process with a tailored PA program in order to improve elementary functional capacities to be physically active. This approach is of utmost importance as the impact of a substantial weight loss on a variety of functional parameters have barely been studied (17, 18). The results of this study might provide further insights in the relevance of an exercise program to accompany a substantial weight loss.

## Method

Subjects with obesity who attended the Unit of Therapeutic Patient Education of the Division of Endocrinology, Diabetes, Nutrition and Therapeutic Patient Education, University Hospital of Geneva were recruited prospectively when participating to educational sessions for bariatric surgery preparation. The study coordinator explained the study protocol and people who were interested to participate received a written information. The study coordinator fixed a first appointment which took place maximum 10 days before the surgical intervention. During this baseline visit, patients’ characteristics and clinical variables were recorded and individuals’ functional capacities assessed. We recorded clinical variables (age, sex, body weight, body mass index (BMI) and waist circumference) which are routinely assessed pre-surgery. Waist circumference was calculated as the sum of three trials. In addition, we tested the quadriceps strength, the walking capacity, spatio-temporal gait parameters, dynamic balance, lower limb power and quality of life.

Participants then underwent their planned bariatric surgery (Roux-en-Y Gastric Bypass) and were contacted 10 weeks post-surgery to schedule a new appointment. During this follow-up visit, that took place three months postoperatively, the same measurements as in the pre-surgery appointment were re-evaluated. Participants did not follow any specific physical activity program during this period.

### Selection criteria

In this prospective observational study, we consecutively included 46 people with obesity, aged ≥ 18 years with a BMI ≥ 35 kg/m^2^ who attended the Unit of Therapeutic Patient Education of the University Hospital of Geneva to undergo a bariatric surgery and agreed to participate in the study. All participants were recruited from April 2017 to March 2019.

We included only people who agreed to participate and who signed the informed consent. We excluded subjects whose French was not sufficient to understand the study information and patients with any other medical conditions than obesity (e.g., cognitive disorders, pathologies of the neurological or locomotor system) that could interfere with the walking capacity or the tests of functional capacities.

#### Primary Outcome

##### Walking Capacity

Walking capacity was chosen as primary outcome as it is a valid index of patient’s ability to perform daily activities (19, 20). The ability to walk a certain distance is linked to quality of life (16) and to mortality (21). Moreover, walking is a recommended activity for people with obesity (22), affected by weight-related physical difficulties (13).

To assess the walking capacity, a 6-Minute Walk Test (6MWT) was performed according to the American Thoracic Society (ATS) guidelines (19). The 6MWT is a simple and powerful method to assess physical capacity of people undergoing bariatric surgery (23).

Participants walked as far as they could during six minutes in a 30m hallway. The walking distance as well as the dyspnea, rated on a Borg scale (24), were recorded.

#### Secondary Outcomes

##### Spatio-Temporal Parameters

Stride length (SL) and gait speed (GS) were assessed when subjects walked on a 30m flat surface first at a usual gait speed and then at a fast speed without running. Individuals were equipped with inertial sensors on their shoes (Gait Up^®^ system) (25). The mean of the right and the left feet were used to calculate gait speed and stride length.

##### Dynamic Balance

The Functional Reach Test (FRT) was used to assess individuals’ dynamic balance. At the beginning of the test, participants stand in an upright position with one arm raised at 90 degrees of shoulder flexion, hand in a closed fist position (26). A mark was made on a yardstick fixed on the wall specifying the position of the third metacarpal and corresponding to position 1 (26). Then participants were asked to bend as far forward as possible without falling or taking a forward step (26). The end position of the third metacarpal was again marked on the yardstick corresponding to position 2. The difference between position 1 and position 2 was then calculated. Every subject realized this test three times and the mean difference over the three trials was retained for further analysis (26).

##### Quadriceps Strength and Lower Limb Power Performance

**Figure 1:**
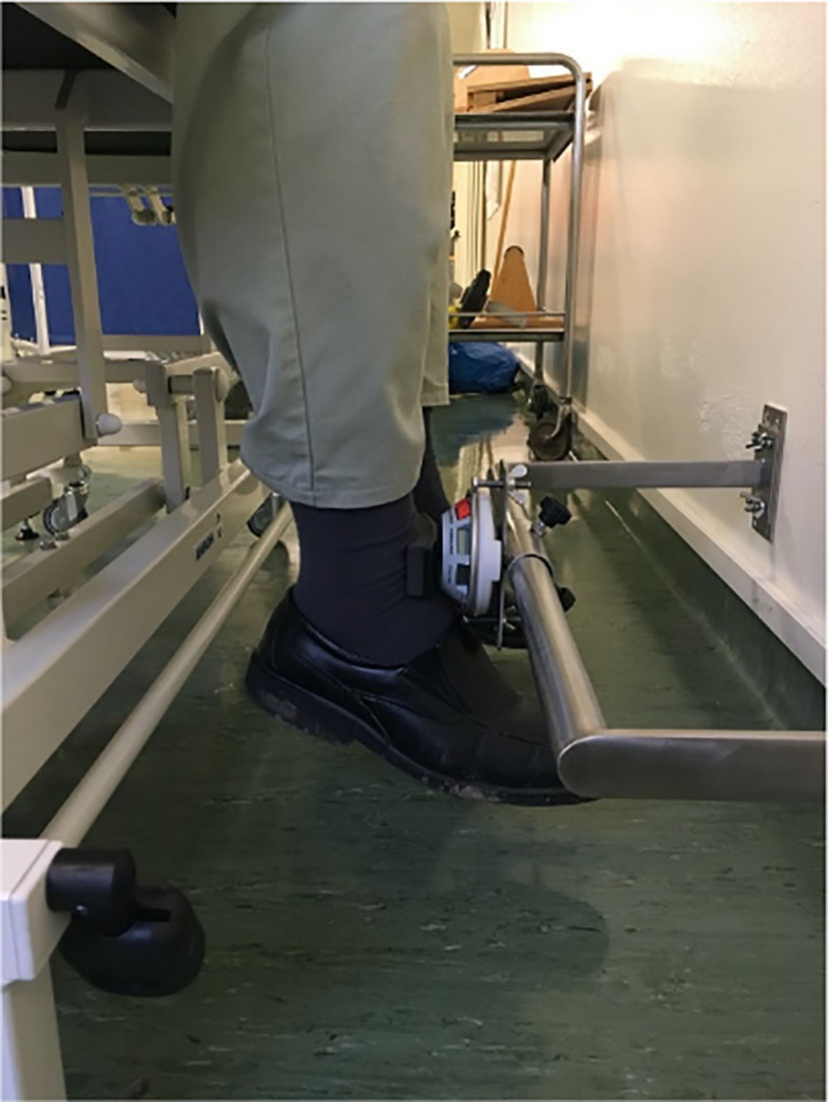
Analytical quadriceps strength assessed in a sitting position, with a dynamometer fixed to a custom-made frame.

The quadriceps strength was assessed with a dynamometer (MicroFet^®^) fixed to the wall on a custom-made frame (**Figure 1**) (27). The lower limb power performance was assessed with the 5 times Sit to Stand test (5STS) (28).

For the quadriceps strength test, the subjects were seated on a treatment table. They were asked to do an isometric knee extension by pushing as hard as possible for five seconds against the dynamometer which was placed at the height of the malleoli and fixed on a custom-made frame (27). The test was repeated three times and the mean of the three trials per leg was retained for further analyses. The normalized strength was then calculated dividing the strength by the weight.

For the 5STS, patients were seated in a chair without armrest with a 90° knee flexion. Subjects were then asked to stand up five times as quickly as possible with the arms crossed on the chest. The time needed to complete the five repetitions was recorded (28).

##### Quality of Life

Participants filled in the Impact of Weight on Quality of Life questionnaire (IWQOL-Lite^©^) created specifically to assess the quality of life of people with obesity (29).

This is a 31-item questionnaire evaluating social, professional, sexual life, self-esteem and mobility. Higher scores are sign of a better quality of life in the domain evaluated (29).

### Statistical Analysis

All analyses were conducted with Stata software (v.15, Stata Corporation, USA). Descriptive statistics were used to summarize characteristics of the study population and to report functional capacities. They were expressed in percentage (sex, comorbidities) or mean ± SD (age, body weight, circumference, BMI, functional capacities and quality of life). To assess the differences before and after surgery, we performed paired t-tests, after checking that data were normally distributed. Linear regressions were used to assess correlations between weight loss and different functional abilities. We performed univariable and multivariable linear regressions. The multivariable model consisted of a dependent variable (difference between pre-post value of functional capacity), a primary exposure factor (weight loss) and co-factors (age, strength difference between pre-post, baseline level of functional capacity). Co-factors were chosen in relation to their clinical interest as potential factors that could have an impact on weight loss and physical function. Application conditions of the regression were verified. The significance threshold was set at 0.05.

#### Determination of Sample Size

Based on a minimal clinically important difference of 20m (range between 14 and 30.5m) of the 6MWT (30) and a standard deviation of 40.3m for people with obesity after surgery (23) with a statistical power of 0.8, an effect size of 0.5 and a probability level of 0.05, 27 subjects were required to complete de study. Based on previous pilot experimentations, we expected a dropout rate of about 40% thus we included 46 participants.

### Ethics

Each participant provided written informed consent after having received information about the study and time to decide about participation. The study was approved by the ethical commission in Geneva (CCER – 2017-00133).

## Results

### Baseline Characteristics

At baseline, 46 subjects, aged 45.46 ± 11.93 years, were assessed. Of the 46 subjects, 14 (27%) were men. The baseline characteristics of the 46 participants are shown in **Table 1**.

**Table 1:** Baseline Characteristics of participants (n = 46).

Characteristics	Value
Age, (years)	44.55 ± 11.41 (25.15-67.78)
Age women	43.25 ± 11.55 (25.15-67.78)
Age men	48.66 ± 10.38 (26.16-61.15)
Sex, n (%)	
Women	35 (76)
Men	11 (24)
Waist circumference, (cm)	129.75 ± 16.07 (96.33-177.75)

Data are presented as mean ± SD (Min – Max). (n = 46).

### Study Completion

Among the 46 subjects, 33 participants (63%) completed the three months follow up. Main reasons for dropouts were subjects refusal to participate because of planning constraints (work) or subjects lost to follow-up (not reachable). No participant announced inconveniences with the procedure or individual tests.

### Changes Observed

All 33 participants who completed the study lost weight (mean weight loss 24.10 ± 6.89 kg, p<*0.001*) and decreased both BMI (-8.58 ± 2.17 kg/m^2^, p<*0.001*) and waist circumference (-14.36 ± 7.89 cm, p<*0.001*) three months after surgery, as expected. As well, quality of life improved significantly (total IWQOL-Lite^©^ score increased by 18.68 ± 14.02%, p<*0.001*) after surgery. The baseline and three-months post-surgery values are shown in **Table 2**.

**Table 2:** Changes in anthropometric parameters, functional capacity, and quality of life pre- to post-surgery (n = 33).

Characteristics	Pre-surgery	3M post-surgery	Difference	P value
***Descriptive parameters***				
Age, (years)	43.57 ± 10.57			
Age women	42.09 ± 9.81			
Age men	48.18 ± 12.17			
Sex, n (%)				
Women	25 (76)			
Men	8 (24)			
Comorbidities, n (%)				
Diabetes	7 (23)			
Smoking	3 (9)			
***Anthropometric parameters***				
Body weight (kg), (n=33)	127.56 ± 27.52	103.46 ± 25.61	-24.10 ± 6.89	<0.001*
BMI, (kg/m^2^) (n=33)	45.43 ± 7.89	36.86 ± 7.83	-8.58 ± 2.17	<0.001*
Waist circumference, (cm) (n=33)	130.15 ± 17.60	115.79 ± 17.56	-14.36 ± 7.89	<0.001*
***Functional capacity***				
6MWT, (m) (n=33)	482.94 ± 61.44	518.21 ± 63.55	35.27 ± 43.48	<0.001*
Gait speed normal, (m/s) (n=32)	1.23 ± 0.14	1.30 ± 0.11	0.08 ± 0.11	<0.001*
Gait speed fast, (m/s) (n=32)	1.61 ± 0.16	1.62 ± 0.16	0.02 ± 0.12	*0.381*
5STS, (sec) (n=33)	9.78 ± 3.63	8.44 ± 2.74	-1.34 ± 2.85	0.011*
FRT, (cm) (n=33)	35.43 ± 6.56	37.39 ± 6.69	1.96 ± 6.06	*0.072*
Strength, (N) (n=31)	281.96 ± 105.28	233.97 ± 73.62	-47.99 ± 67.8	<0.001*
Normalized strength, (N/kg) (n=31)	2.22 ± 0.74	2.29 ± 0.75	0.08 ± 0.58	*0.470*
***Quality of life***				
IWQOL total score, (%) (n=33)	52.68 ± 22.98	71.35 ± 22.71	18.68 ± 14.02	<0.001*

Data are presented as mean ±SD; BMI, Body Mass Index; 6MWT, Six Minutes Walking Test; FRT, Functional Reach Test; IWQOL, Impact of Weight on Quality of Life questionnaire. (n = 33) except for diabetes (n = 31) gait speed normal, gait speed fast and strength (n=32); *p < 0.05.

Individuals improved their mean walking distance during the 6MWT by 35.27 ± 43.48m, p<*0.001*. Normal gait speed and lower limb power were also better after surgery (0.08 ± 0.11m/s, p<*0.001*; -1.34 ± 2.85sec, p=0.011) whereas fast gait speed, dynamic balance and normalized strength did not change significantly after surgery (**Table 2**).

### Univariable and Multivariable Linear Regressions

Univariable analysis shows that for a loss of 1 kg of body weight, the walking distance would increase by 0.73m during the 6-minute walk test. However, the result is not significant which indicates that we cannot be sure that there is a real association between the weight loss and the change in walking distance. The same is true for all other functional capacities except for the normalized quadriceps strength (p=0.048, effect size=0.15), which improves by 0.03 N/Kg per kg of weight loss.

The similar coefficients between the univariable and multivariable analysis indicate that neither age, strength nor the respective functional capacity at baseline influenced the association between the functional capacity and the weight loss (**Table 3**).

**Table 3:** Coefficients of linear regressions using the change value of respective functional capacity as dependent variable and weight loss as primary exposure factor (univariable) and age, strength difference between pre-post, and baseline level of the respective functional capacity as co-factors (multivariable).

Physical performance	Univariable coefficient	IC95%	Effect size	P value	Multivariable coefficient	IC95%	P value
6MWT, (m)	0.733	-1.56 to 3.03	*0.01*	*0.520*	0.790	-1.74 to 3.32	*0.527*
Gait speed normal, (m/s)	-0.001	-0.01 to 0.01	<*0.01*	*0.765*	0.001	-0.01 to 0.01	*0.700*
Gait speed fast, (m/s)	-0.002	-0.01 to 0.00	*0.01*	*0.554*	-0.001	-0.01 to 0.01	*0.736*
5STS, (sec)	0.064	-0.09 to 0.21	*0.02*	*0.391*	0.037	-0.09 to 0.16	*0.535*
FRT, (cm)	-0.010	-0.33 to 0.31	<*0.01*	*0.950*	0.098	-0.24 to 0.43	*0.552*
Quadriceps strength, (N/kg)	0.030	0.00 to 0.06	*0.15*	0.048*	0.031	0.00 to 0.06	0.034*
IWQOL total score, (%)	-0.228	-0.97 to 0.51	*0.01*	*0.535*	-0.128	-0.92 to 0.66	*0.741*

Coefficients of linear regression; concerning univariable analysis: n = 33 for the 6MWT, 5STS, FRT, IWQOL, n = 32 for the Gait speed, n = 31 for the strength; concerning multivariable analysis: n = 31; *p < 0.05.

## Discussion

The results show statistically significant improvements in anthropometric parameters as expected (weight, BMI, waist circumference), as well as quality of life and functional abilities (walking capacity, normal gait speed, lower limb power) three months post-surgery. In contrast, fast gait speed, absolute strength, normalized strength, and dynamic balance did not improve after surgery. Our hypothesis that -some physical functions improve thanks to weight loss alone (balance, gait and walking capacity) but others don’t (muscle strength and standing up from a chair)- could thus only partially be confirmed.

Previous studies already showed that muscle strength rather decreases after massive weight loss induced by bariatric surgery (17, 31, 32). In our study, the enhanced muscle power and the better walking capacity without improved muscle strength (absolute or muscle strength normalized to body weight) might be explained by the fact that carrying less weight facilitates the conduction of daily life activities, due to biomechanical changes (17). Indeed, a massive weight loss would help people to move better or perform PA (17) as the reduction of friction between the thighs or between the arms and the trunk associated with a reduction in extraneous movement (legs and arms swinging closer) would reduce the energy cost of walking (33). However, improved walking capacity was not related to weight loss in this study nor were other functional abilities. They were not associated with weight loss even after the addition of the co-factors as age, strength, and the respective baseline level of the functional capacity. Only normalized strength was associated to weight loss, probably because standardization integrates the notion of body weight. Alba et al. (17) who evaluated changes in physical function before, 6 and 12 months after bypass surgery did also not find any correlation between changes in lean or fat mass or strength and physical performance (17). Our results thus confirm somewhat the results of Alba et al. and indicate that other underlying factors than the loss of weight itself are associated with the improvement of functional capacities, after a bariatric surgery. Some possible explaining factors are now discussed. First of all, physical factors such as a reduction of joint pain or an improvement in cardiovascular fitness are possible explanations that have to be pursued (17). Second, it might be that an improved quality of lean body mass due to a decrease in fat infiltration in the muscles after the massive weight loss (34, 35) could be related to the improved functional capacities although a decrease in absolute strength (17). Indeed, some studies showed that infiltration into intermuscular fat (fat in between muscle fibers) (36) and intramuscular fat (fat within muscle fibers) (37) are associated with poorer physical functions, possibly due to a decrease in myokine secretion and an increase in inflammatory adipokine interleukine-6 (37). And third, psychosocial factors should be investigated as explanatory factors as people may perform better due to a higher self-esteem, which leads to a greater self-empowerment. Indeed, some data show that people report a better quality of life after surgery due to an improvement in body satisfaction, feeling of fatness and body image avoidance (38). Additional studies with more subjects will allow to better define the reasons for post-surgery functional changes.

The fact that fast gait speed and balance did not improve after the weight loss might be explained by the loss in absolute muscle strength as the latter is an important contributor to physical function (31). In this regard, it is likely that a specific muscle training which accompanies the massive weight loss would be beneficial to enhance the progress in functional performances.

Another reason for the non-improvement of balance would be the time needed for the neuromuscular system to adapt to the new body weight (39). Thus, three months may be too short for the proprioceptive system to adapt and to maintain the center of pressure inside the base of support. A study of Benetti (39) showed that even 12 months seemed too short to find an improvement of balance (39) highlighting the length of this process.

In addition to the previously described results, it should be emphasized that although walking ability, normal gait speed and lower limb power improved statistically after bariatric surgery, this was not always a clinically significant change. We found an improvement of -1.34 sec for the 5STS but the minimal clinically important difference (MCID) was found to be at -5 to -7 sec (40). For gait speed, we found an increase of 0.08 m/s but the MCID was found to be at 0.1 to 0.2 m/s (41). Only the change in the 6MWT reached a clinically relevant change (30) which might indicate a progress in cardiovascular fitness of our study participants. These facts stress the importance of an exercise program that should accompany a weight loss program in order to reach not only statistically but also clinically relevant changes in functional performance measures after a massive weight loss which in turn will be beneficial for maintaining the success of lost weight.

A physical program specially based on these factors (gait parameters, strength, and balance) could indeed promote the improvement of functions after weight loss. A pilot study of Stegen et al. (31) showed an enhancement in functional capacity (strength and aerobic capacity) after an exercise training program in the first four months after surgery. People in the control group did not show the same improvements (31). Beyond the changes in function, exercising can also limit the loss of FFM (31) which is crucial as its loss is related to health problems and an increased all-cause mortality (9). A physical activity program seems also necessary to sustain the weight loss over time (7, 12). A qualitative study on the perception of PA after bariatric surgery showed that PA can further help people feel more comfortable with their new body shape and have more energy to accomplish daily household activities (13) which might enhance empowerment. Furthermore, they felt happier and had a better sleep (13).

Even when sending a reminder of the appointment the week before the test and on the morning of the evaluation, we had a relatively high dropout rate (37%). The dropout rate is in accordance with our previous pilot experimentations. However, the dropout rate additionally to a relatively small sample size may have diminished the power of this study even if the sample size had previously been calculated. The small sample size hindered us from adding some co-factors in the multivariable analysis and consider all possible parameters that may influence the relation between a functional capacity and loss of weight. Adding the circumference for the FRT multivariable analysis would thus have been valuable as we know that a forward shift of the center of gravity due to abdominal fat can negatively impact balance (42). Furthermore, the 7% improvement of walking capacity was smaller than the 31.6% found in another study one year post surgery (16). This is showing that improvements in physical ability can occur after three months (17) but a longer follow-up might be better to observe the effects of weight loss on walking capacities.

Futures researches should be conducted with larger sample sizes and longer follow-up. Indeed, body composition as well as functional abilities can vary significantly during the first year after surgery and it is important to see how the patient evolves beyond three months. A physical program added during this follow-up period should also be investigated. Finally, the factors that may explain the improvement in functional abilities after bariatric surgery should be evaluated. Joint pain, cardiovascular fitness, lean mass quality and psychosocial factors such as self-esteem, and self-empowerment should also be assessed.

## Conclusion

While anthropometric parameters, walking capacity, normal gait speed, lower limb power and quality of life improve after surgery, other parameters don’t (fast gait speed, absolute and normalized strength, dynamic balance). This study highlights the fact that some functional abilities don’t progress enough after weight loss induced by surgery. In addition, some others were enhanced but the improvement was not considered as clinically relevant. Adding a physical activity program based on strength and balance might be relevant. It might be considered in future investigations and future practice.

## Data Availability Statement

The raw data supporting the conclusions of this article will be made available by the authors, without undue reservation.

## Ethics Statement

The studies involving human participants were reviewed and approved by Commission Cantonale d’Ethique de la Recherche sur l’être humain (CCER) Genève. The patients/participants provided their written informed consent to participate in this study.

## Author Contributions

AR contributed to the acquisition, to the analysis and interpretation of data and to the writing of the article. SG contributed to the conception of the project, to the recruitment, to the acquisition and interpretation of data and to the revision of the article. RH contributed to the analysis and interpretation of data and to the revision of the article. A-VB contributed to the acquisition, to the analysis and interpretation of data and to revision of the article. ZP contributed to the conception of the project, to the recruitment, to the interpretation of data and to the revision of the article. LA contributed to the conception of the project, to the analysis and interpretation of data, to the coordination and supervision of study process, to the edition and to the revision of the article. All authors contributed to the article and approved the submitted version.

## Conflict of Interest

The authors declare that the research was conducted in the absence of any commercial or financial relationships that could be construed as a potential conflict of interest.
